# The Paipu framework enables creation of a large-scale mammalian cancer transcriptomics atlas

**DOI:** 10.64898/2026.05.14.725161

**Published:** 2026-05-18

**Authors:** Bria S. Smith, Leslie A. Smith, Ji-Hyun Lee, James A. Cahill, Kiley Graim

**Affiliations:** 1 Department of Computer and Information Science and Engineering, University of Florida, 1889 Museum Rd, Gainesville, 32611, Florida, USA; 2 Department of Biostatistics, University of Florida, 2004 Mowry Rd, Gainesville, 32603, Florida, USA; 3 Department of Environmental Engineering Sciences, University of Florida, 365 Weil Hall, Gainesville, 32611, Florida, USA; 4 UF Genetics Institute, University of Florida, 2033 Mowry Rd, Gainesville, 32610, Florida, USA; 5 UF Health Cancer Center, University of Florida, 2033 Mowry Rd, Gainesville, 32610, Florida, USA

**Keywords:** comparative genomics, pan-cancer, pipeline, atlas

## Abstract

A plethora of studies have identified shared molecular mechanisms involved in tumor development across humans and other mammalian species. While these two-species analyses advance understanding of human disease, extending them across many species would provide evolutionary insight into molecular mechanisms driving human cancers. However, this expansion requires knowledge transfer and harmonization across species. Genomic differences between species, including variation in genome annotation quality, have historically hindered multi-species large-scale atlas creation. To overcome these challenges, we present Paipu, a comprehensive pipeline designed to streamline querying, preprocessing, harmonization, and retrieval of large-scale RNA-seq data and associated metadata from the NCBI Sequence Read Archive (SRA). Paipu facilitates multi-species analysis by creating a harmonized atlas from user-defined search terms and species. It consists of three components: reference genome preparation, SRA metadata retrieval, and RNA-seq data processing. We apply Paipu to 188 cancer-related terms in 239 non-human mammalian species, creating a harmonized atlas of 3,484 RNA-seq samples spanning 17 species and 35 cancers. This pan-mammalian pan-cancer atlas enables myriad comparative genomics analyses that leverage genetic variation to better understand rare human cancers. As such, Paipu serves as a resource for cross-species cancer genomics and supports atlas creation for any set of species and search terms.

## Introduction

Advancements in high-throughput sequencing technologies have generated vast amounts of genomic data. As of Jan 01, 2026, >49 million BioSamples have been uploaded to the Sequence Read Archive. More than 8 million of these samples (~16%) were uploaded in 2025 alone. This large-scale data provides the opportunity for cross-species analyses on an unprecedented scale. Unfortunately, differences in reference genomes, gene annotations, and sequencing technologies have, until recently, posed significant barriers to large-scale many-species data harmonization efforts. While databases such as the Sequence Read Archive (SRA) [40] store over 25 petabases of genomic data across more than 14 million sequencing runs [31], processing these data in a manner that allows for cross-species analysis is computationally intensive and requires comparative genomics expertise.

Previous comparative cancer genomics studies involving humans plus one other species have revealed key insights into disease mechanisms and evolutionary relationships. For example, studies have investigated cancer resistance in elephants [4], many spontaneous cancers in dogs [10, 22, 23, 39, 38], fibropapillomatosis in sea turtles [17], and genomic adaptations associated with cancer resistance in naked mole rats [56]. In addition, phylogenetic analyses identified 40 human cancer genes as being under positive selection in mammals [60]. While these studies have accelerated scientific understanding of human cancers, scaling these approaches to larger-scale studies presents significant technical challenges. Low-quality and poorly annotated genome assemblies can result in misalignment and inaccurate ortholog mapping [33, 36, 25, 50]. These and other challenges limit the potential of many-species analyses.

Despite these limitations, the benefits gained from increasing the number of species used in analyses are substantial. Integrating data from a wide range of species enables insights into how millions of years of evolutionary processes have influenced cancer incidence, pinpointing molecular mechanisms of cancer suppression. Increasing the range of genomes used in comparative studies can reveal shared and lineage-specific variation across species, leading to the identification of novel mechanisms of cancer risks and resistance. For example, a cross-species reference genome analysis revealed how life-history traits such as lifespan and body size relate to cancer defenses [48]. Similarly, comparative genomic analyses across 63 mammalian species found that longer lifespan correlates with higher tumor suppressor gene copy numbers, indicating strong cancer defenses in long-lived species such as the naked mole rat [58]. Integrating cancer incidence data across many species, including whales, elephants and Tasmanian devils, can further reveal the molecular mechanisms underlying why certain species are notably vulnerable or resistant to cancer [9]. By leveraging data from many species, researchers can uncover evolutionary insights that may guide cancer treatment and prevention in humans.

There is currently no harmonized repository of mammalian cancer omics data spanning both human and non-human species. However, several notable human pan-cancer datasets have been instrumental in advancing cancer research. Large-scale cancer genomics initiatives such as The Cancer Genome Atlas (TCGA) [62], the International Cancer Genome Consortium (ICGC) [15], and the Pan-Cancer Analysis of Whole Genomes (PCAWG) [3] have generated extensive human cancer genomics datasets across multiple cancer types, including molecular data types such as gene expression, somatic mutations and copy number alterations. The Treehouse Childhood Cancer Initiative [7] is the largest compendium of childhood cancer data and highlights how transcriptomic data can be harmonized across many studies. Together, these resources demonstrate the value of large-scale harmonized cancer datasets.

One barrier to multi-species studies is the lack of cancer incidence and mortality data for most species [14, 61]. Recent efforts have focused on coordinating the sharing of biospecimens and associated data across zoological and conservation communities [30] and developing cross-species data portals such as the Global Genome Biodiversity Network (GGBN) Data Portal [16]. Additional efforts, such as the Arizona Cancer Evolution Center [12], highlight growing interest in cross-species cancer analysis. Both this information and omics datasets spanning many species are essential to enable large-scale comparative cancer genomics across mammals. The lack of a centralized resource encompassing both human and non-human mammalian cancer data presents a significant challenge for multi-species comparative analysis.

Here we present the Paipu pipeline to facilitate the transition of comparative oncology studies from human plus one or two other species to pan-mammalian analyses. Paipu takes as input a list of species and search terms, then finds and retrieves all RNA-seq samples on SRA that fit those search terms. Paipu downloads, processes, and harmonizes metadata and gene expression data for all samples it retrieves. The resultant output is a ready-to-use atlas. We apply Paipu to 239 non-human mammals in the Zoonomia multispecies alignment [2] using 188 cancer-related keywords, creating a pan-mammalian tumor atlas that facilitates cross-species comparative transcriptomics.

## Materials and methods

The Paipu pipeline consists of three phases: (1) genome preparation, (2) SRA metadata retrieval and harmonization, and (3) RNA-seq data processing. In the genome preparation phase, Paipu downloads and preprocesses reference genomes for read mapping. During the SRA metadata retrieval phase, Paipu collects relevant cancer metadata and sequence data. Finally, in Paipu’s RNA-seq data processing phase it uses the FREYA framework [22] to generate RNA-seq count data from the SRA raw read files.

**Fig. 1:**
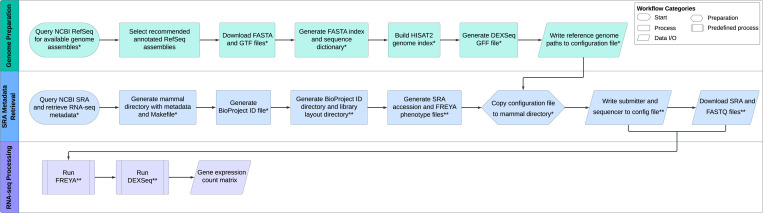
Overview of the Paipu framework. The workflow consists of three main components: reference genome preparation, involving downloading and preprocessing reference genomes for read mapping; SRA metadata retrieval, involving gathering essential metadata and sequence data; and RNA-seq processing via the FREYA pipeline. Asterisks indicate whether steps are performed per mammal (*) or per BioProject (**).

Paipu’s output is organized by species. Each species directory includes a metadata file, Makefile, data processing scripts, BioProject information comma-delimited file, and BioProject subfolders. The BioProject information file includes BioProject IDs, which are unique identifiers for each BioProject defined by SRA. It also includes sample IDs, sequencing layout, instrument platform and submitter organization. Directory subfolders are organized by sequencing layout (single-end or paired-end), each containing FASTQ files for all samples associated with that BioProject and layout type. Additionally, Paipu includes a configuration file, a phenotype file, a file listing sample IDs and RNA-seq count files stored in the corresponding layout subfolders. The Paipu pipeline steps are visualized in Figure 1 and each phase is described in detail below.

### Phase 1: Genome Preparation

For each species, Paipu performs a reference genome preparation phase that retrieves and organizes the required genome and annotation files. Users provide a list of mammalian species names as input. Paipu queries available genome assemblies from NCBI using NCBI Datasets command-line interface [49] and identifies the current recommended reference genome based on assembly release date, annotation availability and associated metadata. It only prepares assemblies that have an associated gene annotation file.

For each assembly, Paipu downloads the genome FASTA and Gene Transfer Format (GTF) annotation files. Once the files are retrieved, the pipeline creates a genome sequence dictionary using Picard [1] and a corresponding FASTA index using samtools [37] to facilitate efficient random access to specific regions of the genome. The pipeline then builds a HISAT2 [32] genome index using the reference FASTA and GTF files to support RNA-seq read alignment. To detect differences in exon usage, it converts the GTF annotation into a General Feature Format (GFF) file using the DEXSeq [5] annotation preparation script. Paipu writes the paths of all generated reference and index files to a configuration file for use in the RNA-seq processing phase.

For species without an NCBI gene annotation, we incorporated TOGA [34] annotations after confirming that chromosome names matched those in the genome FASTA file. Users may incorporate alternative annotations following the same verification.

All reference genome assemblies were obtained from NCBI RefSeq [20] in September 2024 (see Table 1), with the exception of *Bos indicus* and *Tupaia chinensis*, which were obtained in November 2025. For *Enhydra lutris*, the reference genome assembly was retrieved from NCBI, while gene annotations were obtained from TOGA [34]. The reference genomes are organized into distinct mammal directories. Table 1 summarizes the genome assemblies and annotation sources used.

### Phase 2a: SRA Metadata Retrieval

The SRA metadata retrieval phase of Paipu queries the NCBI SRA database [40] to identify RNA-seq samples that match the user-provided species and search terms, retrieves metadata for all matching samples using the NCBI Entrez Programming Utilities (E-utilities) [41] via Biopython [13] (version 1.79), which are subsequently harmonized. Paipu accepts an input file with two columns, one containing a list of species and the other containing a list of search terms. For each species, it queries SRA for each search term provided to retrieve matching metadata. Each query combines the species and search term with built-in parameters specifying the sequencing strategy (rna seq), database name (SRA), and return type (xml). Metadata is retrieved in batches of 500, as recommended by the NCBI E-utilities [41], using Entrez esearch to identify matching records, Entrez read to extract unique identifiers (UIDs), and Entrez efetch to retrieve metadata. Paipu uses a short delay between requests to adhere to NCBI API usage guidelines. It parses retrieved records to extract metadata columns, then cleans and harmonizes the metadata. It then combines data across search terms for each species and remove duplicate records based on run accession.

**Fig. 2:**
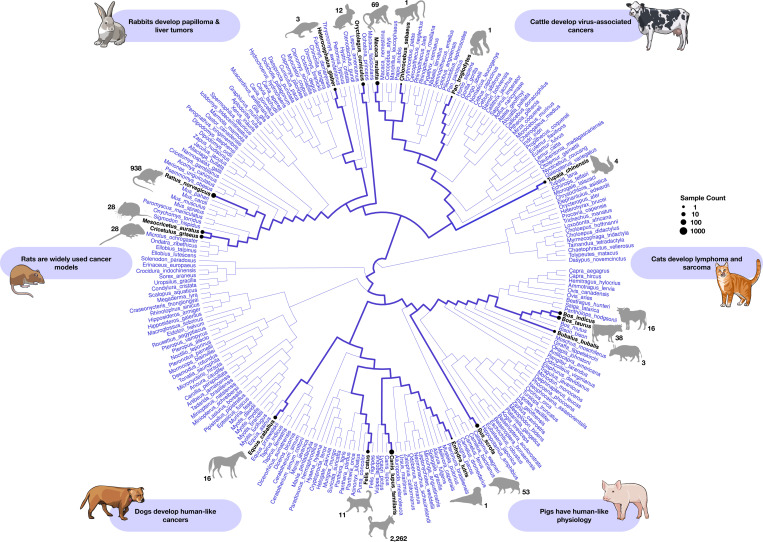
Phylogenetic tree of cancer samples across mammals. The phylogenetic tree of Zoonomia species shows the distribution of cancer samples across species. Black tip labels represent species with cancer samples and purple tip labels represent species without. Point size reflects log-scaled sample counts for species with data. Annotations highlight key cancer types or biological traits in selected species. Animal images were obtained from the NIAID NIH BioArt Source [42, 43, 44, 45, 46, 47].

**Fig. 3:**
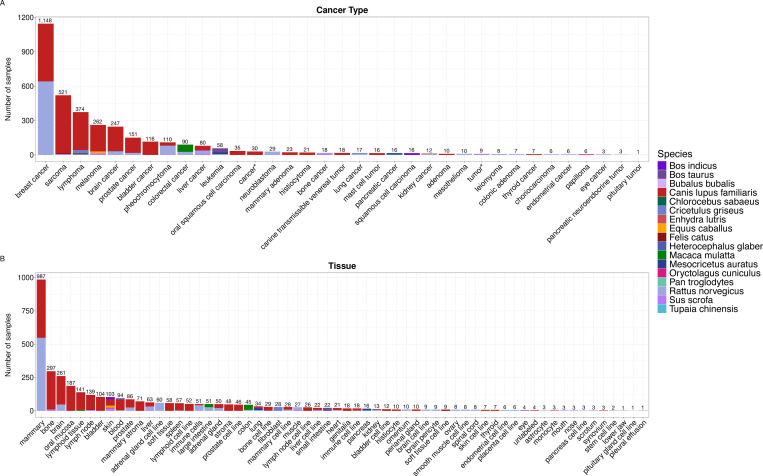
Biological metadata across species. Bar plots show biological and sample composition across species. (A) Cancer type and (B) tissue distributions are shown, where bars represent sample counts and colors represent species. Breast cancer is the most common cancer type observed across *Canis lupus familiaris*, *Rattus norvegicus*, *Tupaia chinensis* and *Bubalis bubalis*. A similar trend appears in the tissue distribution.

We applied this metadata retrieval approach as follows. We used a species list of 239 mammals from the Zoonomia consortium [2] and a manually curated a list of 188 search terms (Supplemental Table S1). These search terms cover a wide range of cancer types from the National Cancer Institute’s (NCI) list of cancer types [27] and the Surveillance, Epidemiology, and End Results (SEER) cancer statistics explorer network [51]. Together, these inputs enable retrieval of detailed metadata on experimental parameters, sequencing platforms, species, tissue types, and tumor classifications. Figure 2 shows a phylogenetic tree including all mammals in the Zoonomia alignment and annotated with tumor samples in the data atlas presented here. Figure 3 summarizes the tumor types and tissues represented in the pan-mammalian pan-cancer atlas.

### Phase 2b: Metadata Harmonization

The metadata harmonization phase standardizes and cleans retrieved sample metadata to ensure consistency across studies. As previously noted [21, 35, 53], there is a non-negligible amount of spelling errors and other discrepancies that complicate harmonizing sample metadata. For example, we identified 5 spelling variants of the term *cell type*. To address spelling issues in the metadata, Paipu uses Levenshtein distance with a threshold of 3 to identify matching columns with minor spelling differences, which are merged into one metadata column. This Levenshtein distance threshold was selected based on unit testing using a variety of thresholds on a sample set of metadata ranging from 2 to 8. We chose a threshold of 3 because it detected the most true positive errors. Overall, this approach improves metadata consistency by reducing redundancy.

We harmonized sequencing strategy by classifying samples as bulk or single-cell based on sample metadata. We labeled samples as single-cell if associated metadata columns contained any of the following keywords: ‘single-cell’, ‘single cell’, ‘scRNA-seq’, ‘scRNAseq’, ‘scRNA’ and ‘singlecell’. We classified all remaining samples as bulk.

After metadata retrieval and initial harmonization by Paipu, we performed additional post-processing steps to further standardize metadata across studies. We standardized common missing metadata value labels (e.g. ‘nan’, ‘unknown’, ‘missing’) to NA across all metadata columns and removed repeated words and duplicated phrases within metadata values. We harmonized several metadata attributes (e.g. cell type, tissue, tumor grade, treatment, and breed) by combining information from related columns identified using keyword and pattern matching. The first non-empty value across these related columns was used to create a single metadata column. Sex labels were standardized by aggregating values across multiple metadata columns and mapping them to MALE, FEMALE, or UNKNOWN. Sequencing platform annotations were standardized, with unspecified values labeled as ‘Unknown’. We assigned tissue annotations using pattern-based matching of metadata values and inferred tissue from cancer type annotations when specific labels were unavailable, ignoring non-tissue terms. Cancer types were further standardized by harmonizing labels and inferring specific cancer types from tissue annotations, when needed. We then grouped cancer types into broader cancer systems using a predefined mapping derived from NCI Cancers by Body Location/System classification [26]. We performed additional curation to correct tissue and cancer type annotations.

As a final step, metadata columns consisting entirely of missing values were removed. These harmonization steps reduced the total number of metadata columns from 263 to 212. All post-processing steps were implemented in R outside of Paipu to allow for specific atlas harmonization without modifying the framework.

### Quality control and filtering

To produce a high-quality atlas, we implemented a methodical process of quality control and filtering. Our original Paipu search found 671,352 relevant samples. Subsequent analysis of these data identified a critical issue in the SRA search function. Samples are returned when a search term appears in any metadata column, including the organization name. As a result, the initial search results included many samples that were unrelated to cancer but were processed at Cancer Centers. To address this, we added a post-search filtering step to the Paipu pipeline. After downloading metadata but before downloading raw reads or processing the samples, Paipu performs a secondary filtering process and removes samples that do not meet the user-provided inclusion criteria. In this post-filtering step, Paipu removes samples in which search terms appear only in metadata columns describing submitter, organization or sequencing center information. Samples are kept if the search term also appears in any other metadata column.

### Data Organization

After downloading and harmonizing the sample metadata, Paipu organizes this information into an easily digestible structure. It then downloads the RNA sequencing reads associated with each SRA accession number in the metadata, in preparation for data processing. To bin samples, Paipu uses the BioProject information file to create a directory for each BioProject. This directory contains subdirectories for ‘single’ and ‘paired’ layout reads. Each subdirectory stores the corresponding SRA accession numbers in a file. Using the SRA accession file, a phenotype file is created in CSV format which contains each SRA accession, a sequentially assigned identification number and a ‘T’ for tumor sample classification for FREYA [22], given that only tumor samples are included.

For the configuration setup, Paipu locates the configuration file associated with the specific mammal and copies it to the mammal’s directory. The pipeline then generates configuration files for each BioProject by populating a copy of the specific mammal’s configuration file with BioProject metadata, including submitter and sequencing platform information. The configuration files are placed in each BioProject’s layout subdirectory for use by FREYA [22] in the RNA-seq processing phase. Paipu then retrieves the raw sequencing data using tools, such as prefetch and fastq-dump, from the NCBI SRA Toolkit [19] to download the SRA files and convert them into FASTQ format. This structured setup enables efficient, organized access to RNA-seq data for each species.

### Phase 3: RNA-seq Processing

The RNA-seq processing phase uses FREYA [22], a framework for RNA-seq data processing and analysis. FREYA performs read alignment using HISAT2 [32], quality control with FastQC [6], and quantifies gene-level expression with DEXSeq-Count [5, 54]. It removes duplicate reads with MarkDuplicates [1], splits and adjusts spliced reads with SplitNCigarReads [59] and assigns reads to alleles with AddOrReplaceReadGroups [1]. For each BioProject layout, the pipeline schedules FREYA to perform read alignment, quality control and expression profile generation. For each sample, FREYA generates high-quality counts files. Once FREYA is complete for a given mammal, Paipu runs DEXSeq [5] to aggregate gene-level counts for each BioProject layout and subsequently generates a count matrix for each. Paipu then combines these counts data into a single gene expression matrix file for each BioProject layout.

### Statistical Analysis of Metadata

We performed statistical tests in R to assess associations between selected metadata variables. Associations between categorical metadata variables were evaluated using Pearson’s Chi-square tests. When contingency tables contained small expected counts (< 5), we estimated p-values using Monte Carlo simulation with 10,000 replicates. We compared quantitative metadata columns across species using the Kruskal-Wallis test. To evaluate the relationship between malignancy prevalence and sample count, we calculated sample counts from our atlas metadata and merged them with malignancy prevalence estimates from Compton et al [14], excluding species without available estimates (n=3). We used a linear regression model with log-transformed sample counts to reduce skewness and additionally assessed monotonic association using Spearman’s correlation. Multiplicity adjustment was performed using the Benjamini-Hochberg false discovery rate procedure.

### Sex Prediction

We predicted sex for samples with missing sex metadata using gene expression from the full joined expression dataset. Gene expression data was normalized using the trimmed mean of M-values (TMM) method in edgeR [55] and transformed to log2 counts per million (logCPM) with a prior count of 1. We defined sex-specific markers using *XIST* as a female marker and a set of Y chromosome genes as male markers, including *DUSP9, CD99, THOC2, SMARCA1, TMSB4Y, RPS4Y1, DDX3Y, EIF1AY, KDM5D, UTY, USP9Y* and *ZFY* [28]. Y chromosome expression was calculated as the sum of expression across these genes for each sample.

We evaluated the predictive performance of *XIST* and Y chromosome gene expression individually using receiver operating characteristic (ROC) analysis on samples with known sex labels and calculated the area under the curve (AUC). Females were defined as the positive class. We then trained a logistic regression model with female sex as the outcome and *XIST* expression and aggregated Y chromosome expression as predictors to evaluate their combined performance.

The combined model had the strongest predictive performance. We applied this model to all samples to obtain the probability of being female and assigned predicted sex labels using a threshold of 0.5. For samples with known sex labels, we kept the original values, but for samples without sex labels, we assigned the predicted labels. We defined prediction confidence based on predicted probabilities, with high confidence for probabilities ≥ 0.9 or ≤ 0.1, medium confidence for probabilities between 0.75–0.9 or 0.1–0.25 and low confidence otherwise.

### Principal component analysis

**Fig. 4:**
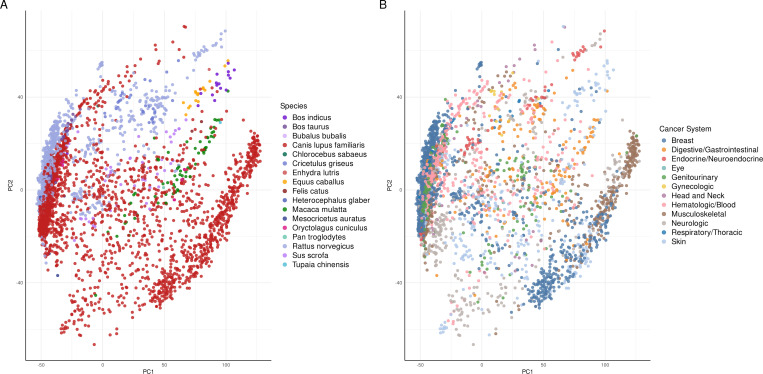
Principal component analysis (PCA) of cancer gene expression across the pan-mammalian pan-cancer atlas. (A) PCA colored by species. (B) PCA colored by cancer system.

We performed principal component analysis (PCA) to explore patterns in cancer gene expression across mammals (Figure 4). For all PCA analyses, we restricted the expression data to only include genes shared across samples. The expression data was then filtered and normalized. Samples with < 2, 600 detected genes were removed (see Supplemental Figure 1), reducing the atlas from 4,112 to 3,935 samples. Genes with low expression were filtered using edgeR [55], with groups defined as the interaction between species and cancer type. After filtering, the gene expression data was normalized using the TMM method in edgeR and transformed to logCPM with a prior count of 1. To reduce redundancy, we calculated pairwise Pearson correlations among samples within each species, and samples with correlation > 0.99 were filtered by removing the sample with the higher mean correlation to other samples (see Supplemental Figure 2). The removal of these redundant samples reduced the atlas from 3,935 to 3,484 samples. We provide both deduplicated and duplicated sample atlases.

We conducted PCA on our deduplicated atlas and visualized results with samples colored by species and by cancer system. We then extended the analysis to include human samples from the Treehouse compendium [7]. We excluded samples with unknown ages and classified human samples as pediatric (age ≤ 18) or adult (age > 18). Genes with zero expression across all samples were removed before normalization. The dataset was then normalized using TMM method in edgeR [55] and transformed to logCPM with a prior count of 1 to be consistent with the processing applied to the deduplicated atlas. The normalized Treehouse and deduplicated atlas datasets were combined by shared genes and batch-corrected between human and non-human samples using ComBat [29]. We then performed PCA on the combined dataset and the results were visualized with samples colored by species and by cancer systems.

## Results

### Pan-mammalian pan-cancer atlas

We used Paipu to create a pan-mammalian pan-cancer atlas from our manually curated list of cancer terms and mammalian species from the Zoonomia multi-species alignment [2]. Human and mouse were excluded from this publication; however Paipu identified >100,000 mouse samples. Paipu identified and retrieved data for 4,753 non-human mammalian cancer RNA-seq samples spanning 20 species. Using the 188 search terms increased samples significantly. For example, Cahill et al [11] identified 1,437 dog tumor samples using the search term ‘cancer’, whereas Paipu identified 2,835 dog tumor samples. Filtering to remove samples in which cancer-related keywords were only present in non-biological metadata columns reduced this atlas to 4,112 samples spanning 19 species. After duplicate removal, this corresponded to to 3,484 samples spanning 17 species (Figure 2) and 8.4 TB of sequencing data, with individual run sizes ranging from 0.03 to 70.9 GB (median 1.7 GB, mean 2.5 GB). Figure 3 shows the cancer types and tissues found for each species. Cetartiodactyla, and Rodentia are the most represented clades in the data atlas, followed by Carnivora and Primates. As of April 30, 2026, 219 species had no cancer related RNA-seq data in SRA.

### Atlas composition

The atlas contains multiple tumor types across species and various sequencing characteristics. Some tumor types are more frequent in certain species; for example, osteosarcoma samples were predominantly from *Canis lupus familiaris* (n=327), with only one sample from another species, which was *Rattus norvegicus*. Overall, breast cancer was the most common tumor type, followed by sarcomas, lymphomas and melanomas (Figure 3A). Breast cancer was most common in *Rattus norvegicus* (n=641), whereas lymphoma was most common in *Canis lupus familiaris* (n=331).

Samples had a median of 29.7 million reads per sample ranging from 0.5 to 618.1 million reads. Sequencing type also differed by species. Single-cell RNA-seq was represented in *Rattus norvegicus* (38.5%), *Heterocephalus glaber* (100%) and *Canis lupus familiaris* (1.4%), whereas all other species were represented by bulk RNA-seq. Sequencing type was significantly associated with library layout (Pearson’s Chi-squared test with Yates’ continuity correction, adjusted *p* = 6.6 × 10^−16^).

Metadata harmonization improved annotation consistency across samples. The number of unique cancer type labels was reduced from 55 to 35 by aggregating related cancer subtypes into broader categories (e.g. sarcoma subtypes), while retaining the original subtype annotations in a separate column. Tissue annotations were combined into a single column through automated harmonization and manual curation. This tissue harmonization step increased the proportion of samples with tissue annotations from 67% to 100%. Additionally, the proportion of samples with missing biological sex annotations decreased from 59% to 0% following prediction from gene expression.

### Principal component analysis and integration

To evaluate variation in the expression data, we performed PCA on the processed RNA-seq data (Figure 4). The samples followed a continuous distribution in PCA. When colored by species, there was species-specific grouping, which is consistent with differences in gene expression across mammals. When colored by cancer system, there was partial grouping of related cancer systems. These results indicate that species is a major driver of variation and that cancer-related signal is also present.

**Fig. 5:**
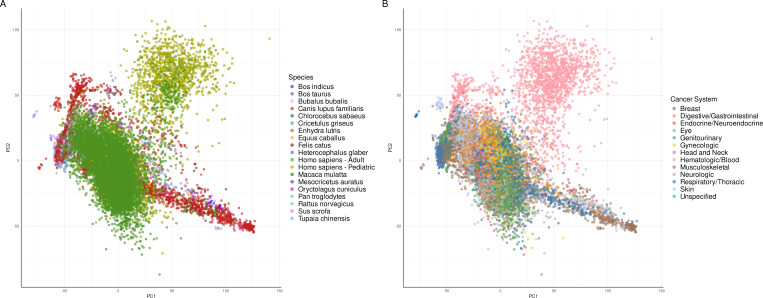
Principal component analysis (PCA) of cancer gene expression across the pan-mammalian pan-cancer atlas and the Treehouse dataset. (A) PCA colored by species. (B) PCA colored by cancer system.

To evaluate integration with external datasets, we integrated the atlas with the Treehouse compendium [7] and performed PCA on the combined dataset (Figure 5). The samples followed a continuous distribution in PCA, with a different structure compared to the atlas alone. Additionally, samples from the pan-mammalian pan-cancer atlas and Treehouse compendium overlapped within cancer systems. These results indicate that the processed expression data is comparable across datasets.

### Individual project enrichment

This pan-mammalian cancer atlas contains data from 200 BioProjects and 3,484 samples. BioProjects have on average 17.4 (median 10.0, IQR 4.8–19.0, SD 30.1), demonstrating the high variability in study sizes. The combined data atlas is 200 times larger than the average BioProject.

### Statistical enrichment in species metadata

**Fig. 6:**
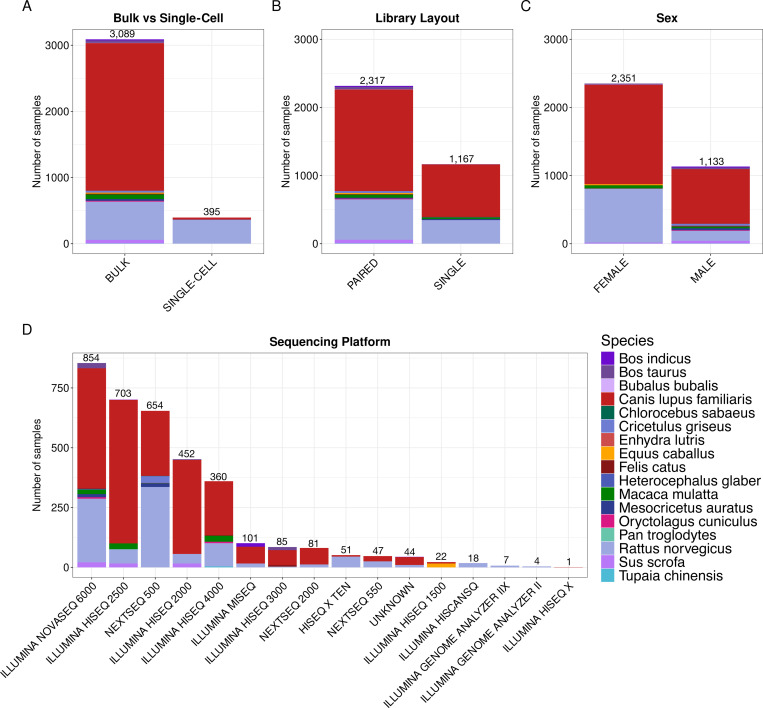
Technical metadata across species. Bar plots show the distribution of key technical and sample attributes across species. (A) Bulk versus single-cell sequencing, (B) library layout, (C) sex, and (D) sequencing platform are shown, where bars represent sample counts and colors represent species. Bulk sequencing and paired-end libraries are most common. Illumina platforms are used for most samples.

The pan-mammalian tumor atlas consists primarily of *Canis lupus familiaris* and *Rattus norvegicus* samples, consistent with expectations given their use in medical and basic science settings. Together, these two species make up 92% of the atlas, with 65% dog and 27% rat samples (Figure 2). Across all species, most samples are bulk RNA-seq (see Figure 6A, 89%). Single-cell RNA-seq data is limited to *Canis lupus familiaris* and *Rattus norvegicus* samples. Most samples are sequencing using paired-end reads (67%), while 33% use single-end reads (Figure 6B). More than 15 unique sequencing platforms are represented in the atlas, with Illumina NovaSeq, HiSeq, and NextSeq used most frequently (Figure 6D).

We then assessed the relationship between cancer type and species using harmonized cancer type categories that aggregate related cancer subtypes. Cancer type was significantly associated with species (Pearson’s Chi-squared test with Monte Carlo simulation, *χ*^2^ = 15, 595, *p* = 1.5 × 10^−4^). Library layout was also significantly associated with species (Pearson’s Chi-squared test with Monte Carlo simulation, *χ*^2^ = 94.19, *p* = 1.5 × 10^−4^), and sequencing reads showed significant differences across species (Kruskal-Wallis test, *χ*^2^ = 160.69, * df* = 16, *p* = 6.6 × 10^−16^).

In contrast, we found no evidence of a monotonic association between malignancy prevalence and sample count across species (Spearman’s *ρ* = 0.02, *p* = 0.95). Linear regression also showed no evidence of a linear association between malignancy prevalence and log-transformed sample count (*slope* = 0.0009, *p* = 0.95), with sample count explaining a very small portion of the variation in malignancy prevalence (*R*^2^ = 0.0004).

### Predicting sex from transcriptomic data

Given that information about biological sex was only available for 41% of samples in the duplicated atlas, we sought to determine the sex of unlabeled samples using computational techniques. Based on results from previous studies [28], we evaluated the predictive performance of *XIST* and aggregated expression of Y chromosome genes for sex classification using samples with known sex labels (1052 female and 547 male). ROC analysis showed that aggregated expression of Y chromosome genes alone achieved an AUC of 0.60, while *XIST* expression alone achieved an AUC of 0.85. A logistic regression model with female sex as the outcome and *XIST* expression and aggregated expression of Y chromosome genes as predictors achieved an AUC of 0.93. Using a probability threshold of 0.5, the combined logistic regression model achieved an accuracy of 88.7%, with sensitivity of 92.2% and specificity of 81.9%. We then applied the logistic regression model to the 2,336 unlabeled tumor samples in the duplicated data atlas. The model predicted 1,584 samples as female and 752 samples as male. After incorporating these predicted labels, the pan-mammalian pan-cancer atlas contains no missing biological sex labels and consists of 67% female and 33% male samples (Figure 6C).

### Pipeline Performance

We ran Paipu on the University of Florida’s HiPerGator high-performance computing cluster using the SLURM scheduler. Processing reference genomes and RNA-seq data and associated metadata across 20 species (4,753 samples) required approximately 1,600 hours of wall time and approximately 38,000 core hours of compute. The compute estimate is based on allocated cores and runtime, rather than actual CPU usage.

## Discussion

Despite the increasing volume of available RNA-seq data, cross-species cancer studies are still limited by the lack of unified datasets that span many species. Here, we developed Paipu, a pipeline that finds and harmonizes SRA data from many species relating to a user-provided list of species and keywords. We apply Paipu to create a pan-mammalian pan-cancer transcriptomic atlas. This resource enables cross-species analyses that were previously difficult due to inconsistent metadata and data being spread across studies.

The pan-mammalian tumor atlas presented here highlights the scale of publicly available transcriptomic data and the challenges associated with integrating many datasets from different sources. Paipu initially identified 4,753 samples across 20 species. After filtering, quality control, and duplicate removal, the atlas size reduced to 3,484 samples spanning 17 species. This reduction in size highlights the importance of quality control when integrating public data. Despite this reduction in size, this atlas still represents a substantial resource. It contains over 8TB of sequencing data across 200 studies. The atlas also reveals strong biases in data availability, as most of the samples are from a small number of species, particularly *Canis lupus familiaris* and *Rattus norvegicus*. For most of the 241 mammals in the Zoonomia alignment, there is no cancer RNA-seq data in NCBI SRA.

Some tumor types are more frequent in certain species, reflecting both biological differences and sampling biases. For example, we found that osteosarcoma samples were predominantly from *Canis lupus familiaris*, which is consistent with its higher incidence rate in dogs compared to humans [11]. In addition to biological differences, sequencing methods also contribute to this data bias. Single-cell RNA-seq data differs from bulk RNA-seq in the amount of information it captures and is often more sparse due to dropout. Dropout occurs when a gene is present in a cell, but is not detected because only small amounts of its mRNA are captured during sequencing [52, 65]. The limited representation of single-cell RNA-seq data across species is likely due to the increased technical complexity of single-cell RNA-seq, higher costs [64], challenges in sample preparation [57] and sensitivity to incomplete annotations, especially in non-model organisms [24]. Only 395 single-cell samples exist in the pan-mammalian data atlas, most of which are *Rattus norvegicus* (rat) tumors. Of these, most (n=363) are breast cancer samples.

Technical differences were observed in sequencing platforms, library layouts and the number of reads per sample. These differences can introduce biases, though approaches such as batch correction may help reduce some of these effects. Some variability is expected, however, given diversity in experimental designs and sequencing strategies across the 200 studies. This highlights the importance of approaches that enable consistent downstream processing of large integrated datasets.

A major challenge in using publicly available data is incomplete and inconsistent metadata annotation [35, 53]. Biological sex information was spread across 6 metadata columns. Paipu harmonization created a global biological sex column using these data, in which 59% of samples were still missing sex labels (n=2,336). To address this, we implemented a logistic regression model based on *XIST* and aggregated Y chromosome gene expression. This model successfully identified biological sex for all samples, resulting in zero missing data for the sample sex column and thus a more complete atlas. This demonstrates how missing information can be deduced from genomic data using computational techniques to address common challenges related to incomplete metadata in public datasets.

While existing tools encompass similar individual Paipu components, they do not provide a comprehensive resource. MetaSRA [8] provides normalized metadata for human samples from the NCBI SRA, addressing challenges in metadata standardization. MetaSRA is, however, limited to human samples. Harmonized data atlases such as Recount3 [63] include more studies than presented here, but these data are restricted to human and mouse samples. The NextFlow nf-core/rnaseq workflow [18] is an alternative for the gene expression data processing and could replace the FREYA pipeline currently used by Paipu. While flexible and well-designed, this workflow does not process metadata.

The absence of a centralized resource that integrates reference genome preparation, metadata retrieval and RNA-seq processing for non-human mammals remains a significant gap in the field. Paipu addresses this gap by enabling consistent processing of RNA-seq data and associated metadata across studies in NCBI SRA. By systematically retrieving and harmonizing public datasets from different studies, Paipu facilitates the use of data that would otherwise remain separate and difficult to compare. It improves the usability of public datasets and facilitates cross-species analyses.

## Supplementary Material

Supplement 1

Supplementary Data

Supplementary data is available at *NAR* online.

## Figures and Tables

**Table 1.** Summary of reference genomes used in the pan-mammalian pan-cancer atlas.

Latin name	Common name	Clade	Ref genome version	Annot source	Protein-coding genes	Genome size (Gb)	Samples
*Bos indicus*	zebu cattle	Cetartiodactyla	GCF_029378745.1	NCBI	20,976	2.7	16
*Bos taurus*	domestic cattle	Cetartiodactyla	GCF_002263795.3	NCBI	21,667	2.8	38
*Bubalis bubalis*	water buffalo	Cetartiodactyla	GCF_019923935.1	NCBI	21,359	2.6	3
*Canis lupus familiaris*	dog	Carnivora	GCF_011100685.1	NCBI	21,175	2.5	2,262
*Chlorocebus sabaeus*	green monkey	Primates	GCF_015252025.1	NCBI	21,620	2.9	1
*Cricetulus griseus*	Chinese hamster	Rodentia	GCF_003668045.3	NCBI	21,776	2.4	28
*Enhydra lutris*	sea otter	Carnivora	GCF_002288905.1	TOGA	19,126	2.5	1
*Equus caballus*	horse	Perissodactyla	GCF_002863925.1	NCBI	20,995	2.5	16
*Felis catus*	domestic cat	Carnivora	GCF_018350175.1	NCBI	20,453	2.4	11
*Heterocephalus glaber*	naked mole-rat	Rodentia	GCF_000247695.1	NCBI	19,978	2.6	3
*Macaca mulatta*	Rhesus monkey	Primates	GCF_003339765.1	NCBI	21,121	3.0	69
*Mesocricetus auratus*	golden hamster	Rodentia	GCF_017639785.1	NCBI	21,616	2.5	28
*Oryctolagus cuniculus*	rabbit	Lagomorpha	GCF_009806435.1	NCBI	22,458	2.8	12
*Pan troglodytes*	chimpanzee	Primates	GCF_028858775.2	NCBI	22,561	3.2	1
*Rattus norvegicus*	Norway rat	Rodentia	GCF_036323735.1	NCBI	23,154	2.8	938
*Sus scrofa*	pig	Cetartiodactyla	GCF_000003025.6	NCBI	20,666	2.5	53
*Tupaia chinensis*	Chinese tree shrew	Scandentia	GCF_000334495.1	NCBI	21,077	2.8	4

## Data Availability

All sequencing data and associated metadata used in this study were obtained from publicly available data from the NCBI Sequence Read Archive. The processed, non-duplicated atlas and the full atlas including duplicated samples are available on Zenodo under https://doi.org/10.5281/zenodo.19904328. Code can be accessed at https://github.com/GraimLab/Paipu.
