# Supplementary material for: The Paipu framework enables creation of a large-scale mammalian cancer transcriptomics atlas
Source: bioRxiv. 2026 May 18:2026.05.14.725161. Preprint. [Version 1] doi: 10.64898/2026.05.14.725161 (PMC13228373; doi:10.64898/2026.05.14.725161)
Supplement: Supplement 1 [file media-1.pdf]

Supplemental Information

Table S1. 239 organisms and 188 cancer-related search terms used as input to Paipu to query and retrieve RNA-seq samples.

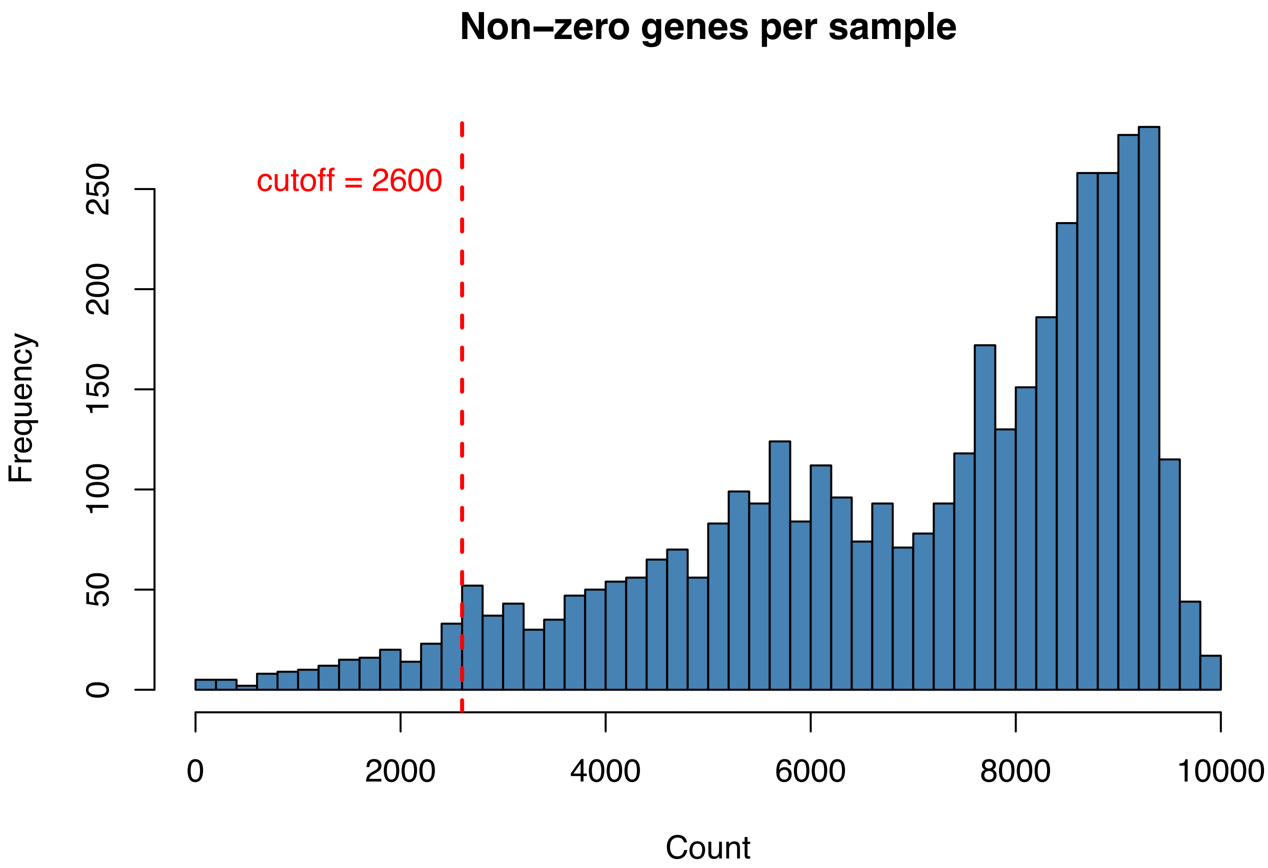

Supplemental Figure 1: **Distribution of expressed genes per sample.** Histogram of the number of expressed genes (non-zero expression) per sample. Samples with $< 2,600$ expressed genes were excluded as low-quality. This cutoff was chosen based on the distribution, with samples below this value forming a left tail of low count samples.

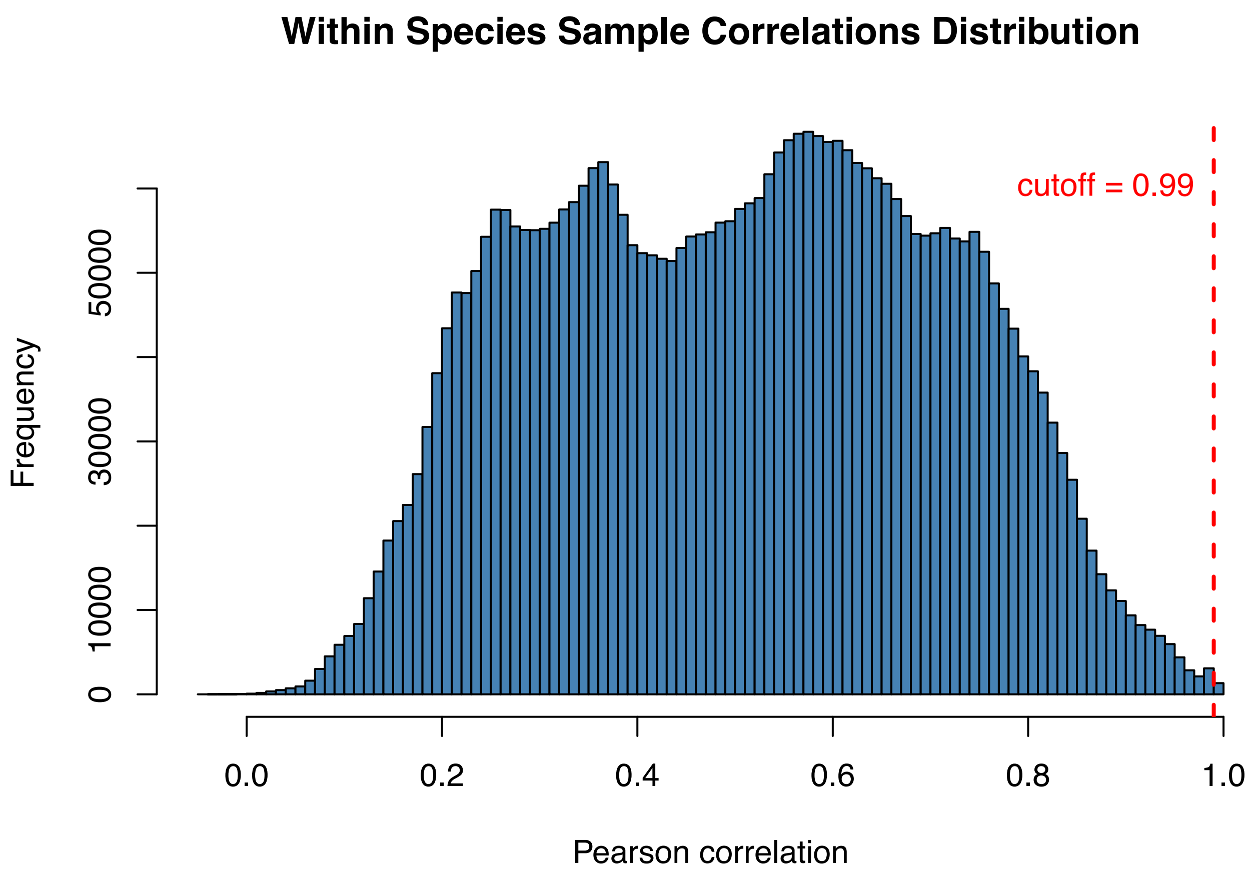

Supplemental Figure 2: **Distribution of within species sample correlations.** Histogram of within species pairwise Pearson correlations. Sample pairs with correlation > 0.99 were considered highly correlated and one sample from each pair was removed based on its mean correlation to other samples.
